# Some Simple Uses of Carbolic Acid. II

**Published:** 1894-06-23

**Authors:** A. G. Miller

**Affiliations:** Lecturer on Clinical Surgery, and Surgeon to the Edinburgh Royal Infirmary


### SOME SIMPLE USES OF CARBOLIC ACID.—II.

By A. G. Miller, M.D., F.R.C.S.E., Lecturer on Clinical Surgery, and Surgeon to the Edinburgh Royal Infirmary.

Some of my students and medical friends having read my former article in this journal (July 1st and 8th, 1893), have asked me to give them more of my experience of the uses of carbolic acid. This I gladly do.

In my former article I gave a history of my acquaintance with carbolic acid from the earliest days of its introduction by Sir Joseph Lister when he was Professor of Surgery in Glasgow. I also described how my attention was first directed to the "antiphlogistic" power of the "carbolic towel"[1] the threefold action of carbolic acid on the skin—astringent, anæsthetic, and antiseptic—and how its great absorbability increases its power and enables it to exert an influence even on the system generally through the circulation.

(*a*) Although I have already given fully my views on the cleansing of the skin of patients and the hands of surgeons, I would like to say a word or two further on one point. I said formerly that a solution of carbolic acid in glycerine "is not so good as a watery solution, because glycerine does not part with the carbolic acid so easily as water does." Mr. G. B. Lockwood[2], on the other hand, seems to consider that "all watery solutions must be inefficacious" for the disinfection of skin, because the latter is always more or less greasy. Theoretically it may be so; but practically I know that carbolic lotion (1 to 20) soaks into and soddens the dirtiest skins. After reading Mr. Lockwood's paper I employed the glycerine preparation for disinfecting the skin of patients before operation, but was not so pleased with it as with the watery solution. For the reason given above I prefer the simple watery solution, but I can imagine that the glycerine compound may be suitable in the case of patients whose skin and kidneys are specially sensitive. In such the reluctance with which the glycerine parts with the carbolic acid may modify and mollify the action of the carbolic acid. In the epitome of the *British Medical Journal* for March 24th, 1894, it is stated that according to Leuti, of Rome, glycerine interferes with the antiseptic action of carbolic acid.

(*b*) In regard to the treatment of erysipelas, I would like to withdraw what I said in my former paper, viz., "that carbolic lotion ought to be useful in some forms of erysipelas." I would rather say that carbolic applications, while they remove the inflammatory appearances, do not check the spread of the disease, and therefore their action is apt to deceive. The best method of treatment is by iodine painting, as described in this journal (October 21st, 1893), by my friend, Mr. Alexander Miles.

(*c*) In inflammatory affections of the cellular tissue, such as whitlow and septic puncture, carbolic lotion (1 to 20) is very useful. If applied soon enough the inflammatory process may be checked, in any case it will be modified.

In this connection permit me to say a word or two on the subject of carbolic lotion *versus* the old-fashioned porridge or linseed poultice. In the days of Liston and Syme poultices were condemned and simple water dressings employed in their stead. These acute observers noticed that poultices increased suppuration and interfered with the healing of wounds. We now know the explanation. These poultices were swarming with microbes, and their warm, moist substance provided an all too favourable culture medium. On the other hand a piece of lint soaked in carbolic lotion (1 to

20), wrapped round a finger, and covered with some waterproof material, is anæsthetic and powerfully antiseptic, as well as warm and comfortable.

I wish I could think that the old, dirty sloppy poultices were a thing of the past! Unfortunately they are not. In the present day many whitlows are made worse and fingers are amputated as the result of poulticing, and I have seen many tubercular glands and joints driven to destruction by the same means. So far as I know, surgery can do without poultices altogether. Aseptic surgery would have a better chance, and hospital surgeons would have much better results were people taught to avoid poultices as an unmitigated evil.

(*d*) A simple traumatic synovitis, or acute bursitis, will get well in a week if the limb be kept at perfect rest; but carbolic lotion (1 to 40) helps, and also relieves pain.

(*e*) Phlebitis, for example, an inflamed varicose vein, is always greatly benefited by carbolic applications. Wrap the limb in a towel soaked with carbolic lotion (1 to 20 or 1 to 40), cover that with some waterproof cloth, and envelope the whole in a domette bandage, and you have an ideal dressing for an inflamed varicose vein. I have tried this in contrast with "lead and opium" and various other applications, and have no hesitation in saying that the carbolic application is the best.

(*f*) Piles.—What a comforter carbolic acid is to a pile! I consider it superior to cocaine or opium. A square of Gamgee tissue soaked in carbolic lotion and applied to an inflamed external pile gives relief at once.

(*g*) Sprains and bruises are soothed, and the swelling reduced by the application of a "carbolic towel" and a firm bandage. I would, however, put this method of treatment second (both as regards time and importance) to ice and massage. Ice for the first twenty-four hours and massage afterwards is the best treatment for a strain or bruise seen early, but when a day or more has elapsed, and there is considerable swelling and discolouration, the carbolic treatment is very useful.

It will be evident to my readers that I recommend the carbolic treatment in cases in which others employ "lead and opium" and evaporating lotions. This is exactly the state of the case, and is the outcome of my observation of the astringent and anæsthetic power of carbolic acid, as already described.

(*h*) Catheters can be cleaned and kept aseptic by carbolic lotion. Let the catheter be well syringed through with 1 to 20 carbolic lotion, and then put into 1 to 40 till wanted. Of course, some catheters are spoiled by being kept a long time in carbolic lotion, especially the gum elastic ones, but an hour of immersion can do no harm. There are many other ways of rendering a catheter aseptic. I mention this one as being very generally applicable and convenient.

(*i*) Internally I have given carbolic acid in three forms in septic conditions. (1) The sulpho-carbolate of soda. I give (as recommended by my friend Dr. Brakenridge), from ten to twenty grains every second or third hour for the first day, and then continue the same dose every fourth or sixth hour. (2) Glycerine and carbolic acid. This combination I used long ago in cases of typhoid fever, giving five drops as a dose (of a 1 in 10 mixture). (3) Quinine and carbolic acid. When I had charge of the erysipelas wards of the old Infirmary (1879-80), I gave the above mixture in a solution, with one drop of carbolic acid to five grains of the sulphate of quinine, and I found the combination very useful. It was also economical, for I found that five grains of quinine in the mixture (which may, perhaps, be a chemical combination—a sulpho-carbolate) brought down the patients' temperature as well as ten grains of the plain quinine would.

I have never found any harm result from the administration of the sulpho-carbolate of soda even in large doses, but the glycerine and quinine compounds produce irritation of the stomach, bowels, kidneys, and bladder, if pushed too far.

This leads me to speak of the risks of carbolic acid applied externally, which I shall do very shortly.

**1. Smoky Urine.**—I have never seen any harm result from this if the carbolic application was at once removed. Some persons seem to be peculiarly susceptible to the irritant action of carbolic acid on the kidneys, children especially, and those with soft, white skins. When a patient cannot stand the "carbolic towel," I apply a towel soaked with corrosive lotion, though I do not think this is quite so reliable for cleansing the skin. The glycerine compound of carbolic acid (1 to 10 or 1 to 20) might be tried.

**2. Erythema.**—I have frequently seen an erythematous condition of the skin from carbolic applications. This, however, does not interfere with the healing of a wound. Eczematous and pustular eruptions from iodoform and mercurial dressings are more common and much more troublesome. A change to boracic dressings is generally sufficient to get rid of all these irritations.

**3. Poisoning.**—I do not think I have traced serious toxic symptoms to carbolic acid, except once in the earliest days of my practice in the case of a child who had empyæma, and whom I dressed "under the spray" daily for some time. The symptoms were those of irritation, gastric, pulmonary, and renal, which, however, all disappeared on the discontinuance of the spray.

I have already referred incidentally to some other drawbacks to the employment of carbolic acid. I would merely remark in conclusion, that, though it has its disadvantages, the more experience I have in the use of carbolic acid the more confidence I have in its applicability to antiseptic surgery.

[1] A towel soaked with carbolic lotion. [2] Brit. Med. Journal, Jan. 27th, 1894.

